# An initial investigation into endothelial CC chemokine expression in the human rheumatoid synovium

**DOI:** 10.1016/j.cyto.2017.05.023

**Published:** 2017-09

**Authors:** Lisa Rump, Derek L Mattey, Oksana Kehoe, Jim Middleton

**Affiliations:** a Keele University and ISTM at Arthritis Research Centre at the Robert Jones and Agnes Hunt Orthopaedic Hospital Foundation Trust, Oswestry, Shropshire, United Kingdom; b Haywood Rheumatology Centre, Haywood Hospital, Burslem, Stoke-on-Trent, United Kingdom; c School of Medicine and ISTM, Keele University, United Kingdom; d Faculty of Health Sciences, School of Oral and Dental Sciences, University of Bristol, Lower Maudlin Street, Bristol, United Kingdom

**Keywords:** Chemokine, Endothelial cell, CCL, Rheumatoid arthritis

## Abstract

Rheumatoid arthritis (RA) is a destructive and chronic autoimmune inflammatory disease. Synovial inflammation is a major feature of RA and is associated with leukocyte recruitment. Leukocytes cross the endothelial cells (ECs) into the synovial tissue and fluid and this migration is mediated via a range of chemokines and adhesion molecules on the ECs. As important mediators of leukocyte extravasation, a number of chemokines from each of the chemokine families have been established as expressed in the RA joint.

However, as little information is available on which chemokines are expressed/presented by the ECs themselves, the purpose of the study was to ascertain which of the CC chemokines were localised in RA ECs.

Immunofluoresence was used to assess the presence of the CC-family chemokines in RA synovial ECs using von-Willebrand factor (VWF) as a pan-endothelial marker and a range of human chemokine antibodies. The percentage of VWF positive vessels which were positive for the chemokines was determined. The presence of the four most highly expressed novel chemokines were further investigated in non-RA synovial ECs and the sera and synovial fluid (SF) from patients with RA and osteoarthritis (OA). Statistical analysis of immunofluorescence data was carried out by Student’s *t*-test. For analysis of ELISA data, Kruskal-Wallis ANOVA followed by Dunn’s multiple comparison test was utilised to analyse differences in sera and SF levels for each chemokine between RA and OA. Spearman rank correlations of sera and SF chemokine levels with a range of clinical variables were also performed.

Chemokine detection varied, the least abundant being CCL27 which was present in 8.3% of RA blood vessels and the most abundant being CCL19 which was present in 80%. Of the 26 chemokines studied, 19 have not been previously observed in RA ECs. Four of these novel chemokines, namely CCL7, CCL14, CCL16 and CCL22 were present on ≥60% of vessels. CCL14 and CCL22 were shown to be increased in RA ECs compared to non-RA ECs, *p* = 0.0041 and *p* = 0.014 respectively. EC chemokines CCL7, CCL14, CCL16 and CCL22 also occurred in RA synovial fluid and sera as established by ELISA. CCL7 was shown to be significantly increased in sera and SF from RA patients compared to that from osteoarthritis (OA) patients (*p* < 0.01), and to have a highly significant correlation with the level of anti-CCP (R = 0.93, *p* = 0.001). Less abundant chemokines shown to be present in RA ECs were CCL1-3, CCL5, CCL10-13, CCL15, CCL17, CCL18, CCL20, CCL21 and CCL23-28.

In conclusion, this initial study is the first to show the presence of a number of CC chemokines in RA ECs. It provides evidence that further validation and investigation into the presence and functionality of these novel chemokines expressed at RA synovial ECs may be warranted.

## Highlights

• Comparison of the presence of 26 of the CC-chemokines in RA synovial ECs.
• The chemokines CCL7, CCL14, CCL16 and CCL22 were established as being present at RA synovial ECs for the first time.
• CCL8, CCL14, CCL19 and CCL22 are significantly increased in RA compared to non-RA.
• Synovial fluid CCL7 may be a novel RA disease marker.

## 1 Introduction

There are currently around 48 chemokines grouped according to structural criteria. Each is a single polypeptide chain of 70–100 amino acid residues which share 20–95% sequence homology, including a number of conserved cysteine residues. The cysteine residues have been utilised in the nomenclature system and give four distinct chemokine subgroups [1], [2], [3]: CC, CXC, C (or XC) and CX3C chemokines. These are further split into:

1. Inflammatory chemokines, such as CXCL8 and CCL2 which are usually only expressed under inflammatory conditions, and as such are found in high levels in the RA joint. Their production can be induced in response to stimulation by pro-inflammatory cytokines such as IL-1 and TNFα [4] and they are important mediators in the recruitment of effector cells of both the innate and adaptive immune system.
2. Constitutive (homeostatic) chemokines, which are expressed continuously, and direct essential physiological processes such as haematopoiesis [5], lymphocyte and dendritic cell homing [6], [7] and the normal immune surveillance of body tissues. Unlike inflammatory chemokines they usually bind to specific single receptors [5].
3. Dual function chemokines, which are involved in normal immune defense and are upregulated in inflammatory conditions. This group includes CXCL9, CXCL10, CXCL11, CCL1, CCL20 and CCL25 [7], [8].

Chemokines stimulate leukocyte recruitment into inflamed tissue. These mediators are bound by ECs and presented to chemokine receptors on leukocytes in the blood leading to leukocyte extravasation into the affected tissue [9]. Much work has been undertaken on chemokines in RA [10]. They have been identified in synovial tissue, cartilage and SF, and are produced by cells such as macrophage and fibroblasts. These chemokines are biologically active and stimulate leukocyte migration. Blocking chemokines and their receptors in animal models of RA have led to reduced severity of disease and significant therapeutic effects. Thus, they have been favoured therapeutic targets of the pharmaceutical and biotech industry with clinical trials carried out using anti-chemokine antibodies and chemokine receptor antagonists. Most of these agents did not show clinical efficacy in clinical trials in RA patients with little exception [11], [12]. One reason for the lack of success in clinical studies may be because many chemokines have been identified in RA joints and it can be problematic to choose the most effective ones to target. Our approach has been to try and identify which chemokines are presented by ECs in RA. Therefore, the main purpose of this study was to report on the presence of the CC-family chemokines in ECs of the rheumatoid synovium, and to identify any highly expressed chemokines which are found at significantly higher levels than in non-RA. We also wished to determine whether highly expressed CC chemokines in the synovium were also present in the sera and/or synovial fluid, and whether they showed any relationship with clinical variables. Differing chemokine profiles for arthritides may allow for the further identification of potential disease markers [13], [14]. The current study compares CCL7, CCL14, CCL16 and CCL22 in RA and OA SF and sera and analyses their correlations with various clinical variables.

## 2 Materials and methods

### 2.1 Ethics

Ethical approval was obtained from the Birmingham and Solihull Research Ethics Committee (reference 11/WM/0035) and patients provided written informed consent.

### 2.2 Synovial tissue and sera

RA knee synovial tissue and SF were obtained from patients who fulfilled the American College of Rheumatology (ACR) criteria for RA. The patients were undergoing joint replacement surgery or synovial effusion removal at the Robert Jones and Agnes Hunt Orthopaedic Hospital, Oswestry (n = 8). Patients had a mean age of 66 years and mean disease duration of 23 years at the time of surgery.

Non-RA control tissue from knee joints (n = 6) was obtained by needle biopsy during outpatient exploratory procedures where arthritis had been excluded as a diagnosis. Histology of RA synovia showed classic synovitis with lymphocyte and macrophage infiltration of the sublining, and lining layer (intima). Non-RA synovia showed comparatively little or no leukocyte infiltration. Synovial tissue samples were taken from the suprapatellar pouch and the medial gutter and placed in Hank’s Balanced Salt Solution (HBSS) for transport to the laboratory.

Paired RA SF and serum samples were taken from a further 17 RA patients and 7 OA patients. A wide range of clinical variables were assessed in these arthritis patients. These included erythrocyte sedimentation rate (ESR), joint scores and disease activity scores (DAS44) [15] to assess systemic inflammation and overall disease activity. Further variables such as early morning stiffness (EMS), grip strength and Health Assessment Questionnaire (HAQ) [16] were used to assess pain and loss of function at the different disease stages, while the amount of joint damage was assessed by the damage scale of the Overall Status in Rheumatoid Arthritis (OSRA) [17]. This has been correlated with articular damage as determined by radiographs using the Larsen score.

#### 2.2.1 Immunofluorescence labelling of synovial tissue sections

Tissue samples were snap frozen in *iso*-pentane (cooled in liquid nitrogen) and then stored in liquid nitrogen. 5–6 μm thick serial cryostat sections of the tissue were cut then air dried at room temperature before being stored at -80 °C. Sections were stained as previously described [18]. Briefly, sections were blocked then incubated for 1 h in the primary antibodies, the working concentrations used were: anti-human mouse monoclonal CCL2 (10 µg/ml; MAB2791), mouse monoclonal CCL3 (10 µg/ml; MAB270), goat polyclonal CCL4 (10 µg/ml; AF-271-NA), goat polyclonal CCL5 (15 µg/ml; AF-278-NA), mouse monoclonal CCL11 (15 µg/ml; MAB320), goat polyclonal CCL16 (15 µg/ml; AF802), goat polyclonal CCL17 (10 µg/ml; AF364), mouse monoclonal CCL19 (20 µg/ml; MAB361), goat polyclonal CCL20 (10 µg/ml; AF360), goat polyclonal CCL21(15 µg/ml; AF366), goat polyclonal CCL24 (15 µg/ml; AF343), goat polyclonal CCL26 (10 µg/ml; AF653), mouse monoclonal CCL27 (20 µg/ml; MAB367), (all R&D Systems, UK), goat polyclonal CCL8 (2 µg/ml; Sc-1307), goat polyclonal CCL13 (4 µg/ml; Sc-9655), mouse monoclonal CCL14 (2 µg/ml; Sc-28388), goat polyclonal CCL18 (4 µg/ml; Sc-9781), goat polyclonal CCL22 (4 µg/ml; Sc-12285), goat polyclonal CCL23 (4 µg/ml; Sc-12263), goat polyclonal CCL25 (2 µg/ml; Sc-12277) and goat polyclonal CCL28 (4 µg/ml; Sc-27339) (all SantaCruz Biotechnology Inc UK), mouse monoclonal CCL1 (2.5 µg/ml; LS-C4342) and rabbit polyclonal CCL7 (2.5 µg/ml; LS-B930) (LifeSpan Biosciences, UK), rabbit polyclonal CCL10 (4 µg/ml; Orb13568), rabbit polyclonal CCL12 (2 µg/ml; Orb132384), rabbit polyclonal CCL15 (4 µg/ml; Sc-28388) (Biorbyte, UK), rabbit anti-human von Willebrand Factor (VWF) (3 µg/ml; A0082) and mouse anti-human VWF (4 µg/ml; M0616) (Dakocytomation, UK). Isotype matched control antibodies were used throughout. Sections were then rinsed for in PBS for 3 × 5 min prior to incubation for 45 min in the secondary antibodies which were *anti*-mouse, rabbit or goat Alexa fluor 488 (3.3 µg/ml) and anti-mouse/rabbit/goat Alexa fluor 594 (6.6 µg/ml) in PBS (Invitrogen, UK). (for specific dilution and blocking buffers used for each antibody please refer to supplementary data table).

#### 2.2.2 Sampling of tissue sections and calculations

For each individual the first 15 blood vessels positive for VWF in 4 fields of view per section were counted randomly and blind (magnification X20), any of those vessels also positive for the chemokine under investigation were then counted.

From this the percentage of chemokine positive vessels was calculated as follows: number of VWF and chemokine dual positive vessels ÷ number of VWF positive vessels × 100. Means were then calculated with standard errors. Immunofluorescence was visualised on a light microscope (Olympus IX51) and yellow staining indicated strong colocalisation between VWF and the chemokine.

### 2.3 ELISA

ELISA was performed using CCL7, CCL14, CCL16 and CCL22 DuoSet ELISA kits (R&D Systems, UK); wash buffer, reagent diluent, substrate solution and stop solution (all R&D Systems) were used throughout according to the manufacturer’s instructions. Titrations were performed to establish the optimal working concentrations for both the serum and SF samples. Sample standards were run for each experiment.

### 2.4 Statistical analysis

For analysis of immunofluorescence Student’s *t*-test was used to compare the mean percentage (±SE) of VWF+ vessels stained with the four most highly presented novel chemokines in RA and non-RA synovia. For analysis of ELISA data, Kruskal-Wallis ANOVA followed by Dunn’s multiple comparison test was used to analyse differences in median sera and SF levels for each chemokine between RA and OA. Spearman rank correlations were performed to assess sera and SF chemokine levels with the range of clinical variables mentioned previously. Statistical analysis was performed using NCSS software (NCSS, Kaysville, UT, USA) with *p* < 0.05 being deemed as significant.

## 3 Results

### 3.1 Abundant chemokines identified as novel in RA synovial ECs

**Fig. 1:**
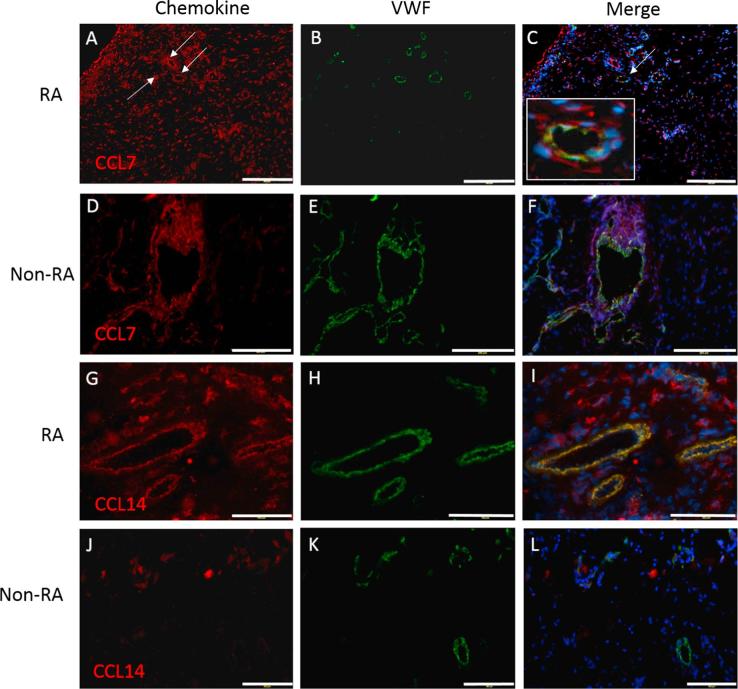
CCL7 and CCL14 in RA and non-RA synovium. For RA CCL7 (A, B, C) and CCL14 (G, H, I), non-RA CCL7 (D, E, F) and CCL14 (J, K, L) chemokines are shown in red with von-Willebrand factor (VWF) in green and DAPI in blue, with merged images to illustrate colocalisation. The white box in Image C shows an enlargement of the vessel indicated by a white arrow in the same image. Scale bars in A-F show 200 µm and in G-L 100 µm. (For interpretation of the references to colour in this figure legend, the reader is referred to the web version of this article.)

**Fig. 2:**
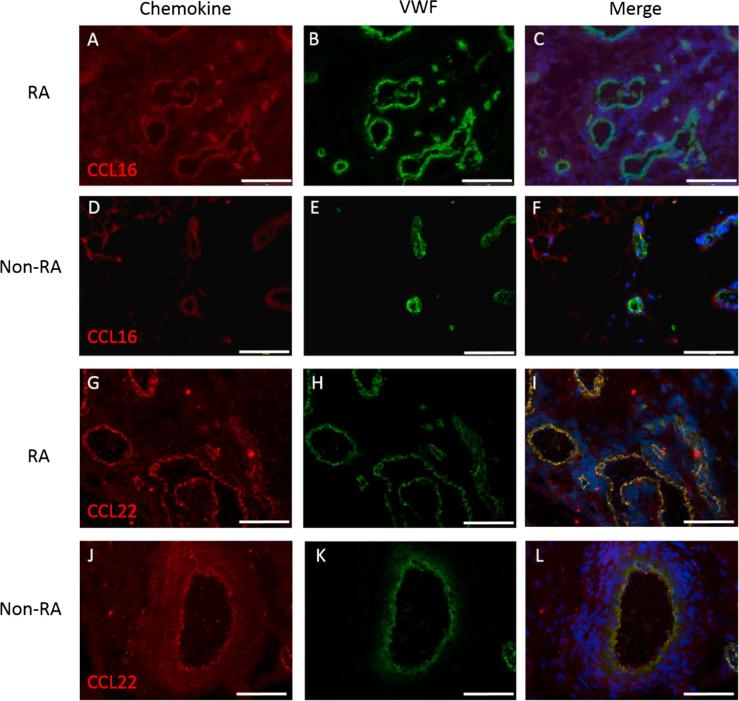
CCL16 and CCL22 in RA and non-RA synovium. For RA CCL16 (A, B, C), CCL22 (G, H, I) and non-RA CCL16 (D, E, F), CCL22 (J, K, L) chemokines are shown in red with von-Willebrand factor (VWF) in green and DAPI in blue, with merged images to illustrate colocalisation. Scale bars in A-C and G-I are 200 µm, and in D-F and J-L 50 µm. (For interpretation of the references to colour in this figure legend, the reader is referred to the web version of this article.)

The chemokines CCL7, CCL14, CCL16 and CCL22 were identified as abundant in RA ECs for the first time. To further assess the importance of these chemokines in RA they were also analysed in non-RA synovial tissue (Table 1, Fig. 1, Fig. 2).

**Table 1:** Most abundant novel chemokines present in VWF positive vessels in RA compared to non-RA synovia.

Chemokine	RA	Non-RA	*p*
CCL7	69.3% (±6.1)	61.9% (±14.8)	*p* = *0.54*
CCL14	73.0% (±7.0)	28.4% (±8.2)	*p* = 0.0041***
CCL16	74.1% (±7.2)	75.3% (±7.0)	*p* = *0.89*
CCL22	60.1% (±8.1)	18.7% (±5.7)	*p* = 0.014*

Data show the percentage of VWF positive vessels that were chemokine positive in RA (n = 8) and non-RA synovia (n = 5). Data are means ± SE, significant differences as assessed by Student’s *t*-test are shown.

* *p* < 0.05

*** *p* < 0.005.

**CCL7**: CCL7 was observed throughout the RA synovium on vessel ECs as indicated by white arrows (Fig. 1A–C), and on stromal cells which appeared in many cases to be cells resembling fibroblasts. A high degree of colocalisation between VWF and CCL7 on vessel ECs was observed (Fig. 1A–C), occurring on 69.3% of vessels in RA. The difference in CCL7 between RA and non-RA tissue (Fig. 1D–F) was not significant (*p* = 0.54, Table 1).

**CCL14:** Very strong staining for colocalisation of CCL14 and VWF (Fig. 1I) was seen in the RA vessels (at 73.0%) (Table 1). CCL14 blood vessel staining was present in the non-RA synovium but was markedly weaker, being present at 28.4% of VWF+ vessels. The chemokine was shown to be significantly increased in RA blood vessels compared to non-RA (*p* = 0.0041, Table 1).

**CCL16:** In RA synovium a high degree of CCL16 staining was seen in vessels with minimal infiltrate staining (Fig. 2A–C). CCL16 was not significantly different when RA and non-RA tissue (Fig. 2D–F) were compared (*p* = 0.89, Table 1).

**CCL22:** In the RA synovium CCL22 colocalisation with VWF+ vessels was strongly evident (Fig. 2G–I). In non-RA CCL22 was observed in VWF+ vessels (Fig. 2J–L) to a lesser degree than in RA synovium. Further to this, the majority of the staining in the non-RA synovium was observed in stromal cells in close proximity to large vessels. CCL22 was significantly increased in RA with 60.1% of vessels being positive compared to 18.7% in non-RA (*p* = 0.014, Table 1).

For all chemokines no background staining was observed on the negative control sections treated with isotype matched control antibodies in place of primary antibodies.

### 3.2 Additional chemokines assessed in RA synovial ECs

The major 4 EC chemokines we concentrated on were CCL7, 14, 16 and 22 (Table 1, Fig. 1, Fig. 2). However in the same samples a further 22 chemokines were observed in RA ECs by dual label immunofluorescence (images not shown) and these are quantified in Table 2 for comparison.

**Table 2:** Chemokines present at VWF positive vessels in RA synovia.

Chemokine	RA
CCL1♣	11.9% (±4.0)
CCL2	51.5% (±6.9)
CCL3	27.4% (±6.1)
CCL4	62.3% (±11.0)
CCL5	44.9% (±7.6)
CCL8	64.1% (±7.4)
CCL10♣	52.6% (±10.0)
CCL11♣	15.8% (±6.3)
CCL12♣	9.6% (±3.2)
CCL13♣	56.6% (±7.0)
CCL15♣	47.3% (±6.3)
CCL17♣	17.4% (±10.6)
CCL18♣	38.0% (±6.4)
CCL19	80.0% (±4.5)
CCL20♣	18.5% (±2.7)
CCL21	36.1% (±10.4)
CCL23♣	37.7% (±4.5)
CCL24♣	28.8% (±4.6)
CCL25♣	28.2% (±4.5)
CCL26♣	59.8% (±7.5)
CCL27♣	8.3% (±1.2)
CCL28♣	40.9% (±6.8)

Data show the percentage of VWF positive vessels in RA synovia (n = 8) that were also chemokine positive.

♣ Indicates novel identification on synovial ECs at the time of writing. Data are means ± SE as a percentage of VWF positive vessels also stained with the chemokine.

**CCL1:** CCL1 was shown to be present in low abundance on both ECs and infiltrates within the RA joint (Table 2).

**CCL2:** CCL2 was present in both infiltrates and ECs. Particularly intense EC staining was observed in regions of more diffuse leukocyte infiltration, with weaker EC staining seen in regions of lymphoid follicles and more dense infiltration.

**CCL3:** CCL3 was observed in RA ECs and predominantly seen on the basement membrane.

**CCL4**: CCL4 was shown to be well represented on RA ECs (62.3% ±11.0) with positive cells also observed in the infiltrate. CCL4 staining was also observed within EC vessel walls in non-RA synovial tissue (49.2% ±16.0) but was not statistically significant compared to RA (*p* = 0.49).

**CCL5:** CCL5 staining was evident throughout the synovium, including EC basement membrane, luminal EC surface, and on infiltrating cells including those within lymphoid aggregates.

**CCL8:** CCL8 was present throughout the RA synovium at 64.1% (±7.4) and the non-RA synovium at 25.6% (±17.0) (*p* = 0.04). Strong colocalisation was seen in VWF+ vessels, while CCL8+ staining of other cell types appeared to be primarily in fibroblast-like cells. .

**CCL10:** CCL10 was shown to be colocalised on VWF+ vessels and also present on infiltrated cells, with particularly intense staining at the synovial intima.

**CCL11:** A number of small VWF+ vessels showed weak staining for CCL11. However, the staining was more intense in larger vessels where leukocyte staining was also present.

**CCL12:** CCL12 stained sparsely and very weakly in VWF+ vessels with a small degree of infiltrate staining also seen.

**CCL13:** Colocalisation between CCL13 and VWF was observed with numerous infiltrating cells also CCL13+.

**CCL15:** CCL15 staining was seen in numerous VWF+ vessels with strong staining in the cells of the intima. Infiltrate staining was seen to be primarily in cells near the vessels or intima.

**CCL17:** Only weak staining of CCL17 could be found on a relatively low number of vessel ECs with infiltrating cells also being CCL17+.

**CCL18:** Leukocyte staining was primarily seen to be on cells in close association with CCL18+ vessels.

**CCL19:** Colocalisation between CCL19 and VWF was observed in RA vessels. Infiltrating cells were also positive for CCL19 particularly in lymphoid follicles with their associated ECs. In the non-RA synovium CCL19 was present, but only on a small number of VWF+ vessels. In RA the chemokine occurred on the ECs of 80.0% (±4.5) of blood vessels and only 10.3% (±2.6) of blood vessels in non-RA (p = <0.0001).

**CCL20:** Very little CCL20 could be found on the vessel ECs but was identifiable in the intimal layer.

**CCL21:** EC CCL21 was observed throughout the synovium and immunoreactivity was noticeable in infiltrating cells.

**CCL23:** A combination of both strongly and weakly positive vessels was seen in the RA synovium. CCL23+ infiltrating cells were also observed which were localised within aggregates.

**CCL24:** CCL24 was observed to localise to both large and small vessels. However, more intense staining was seen on small vessels.

**CCL25:** CCL25 was present primarily on the ECs of larger VWF+ vessels. Where staining occurred on smaller vessels it was seen to be much weaker.

**CCL26:** Colocalisation of CCL26 and VWF was observed in a relatively large number of vessels. Infiltrate staining was also present throughout the samples with some areas being particularly intensely labelled.

**CCL27:** Immunoreactivity was present in infiltrating cells with very few examples of CCL27 and VWF colocalisation occurring.

**CCL28:** Colocalisation between CCL28 and VWF was evident with weaker CCL28 staining observed in smaller vessels. A high degree of intima staining for CCL28 was observed.

For all chemokines no background staining was observed on the negative control sections treated with isotype control IgGs instead of primary antibodies.

### 3.3 CCL7, CCL14, CCL16 and CCL22 in RA and OA SF and sera

**Fig. 3:**
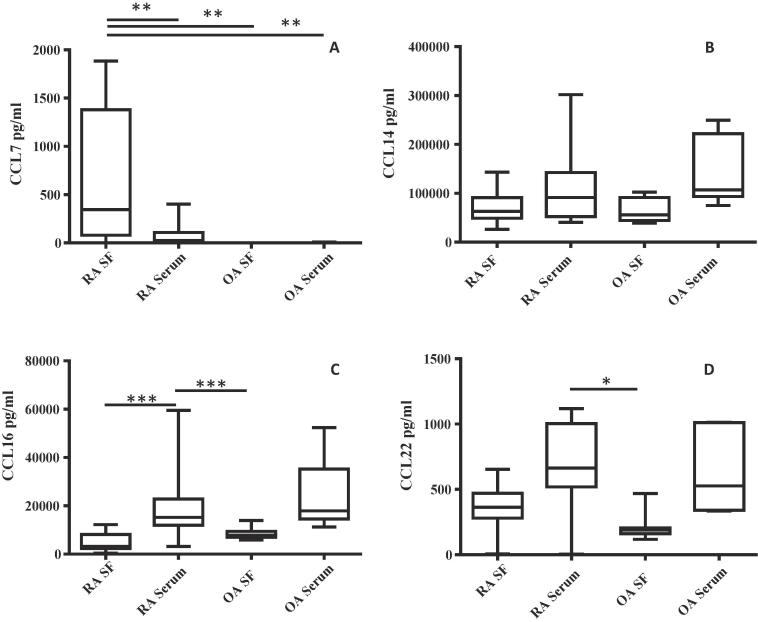
CCL7, CCL14, CCL16 and CCL22 comparisons for RA and OA SF and serum. (A) shows CCL7 levels to be higher in RA SF compared to OA SF, RA serum and OA serum at *p* < 0.01. (B) shows the presence of CCL14 in RA and OA. (C) shows CCL16 to be higher in RA serum compared to RA SF and OA SF at *p* < 0.001.(D) shows CCL22 as increased in RA serum compared to OA SF at p < 0.05. The vertical bar indicates overall range, the box shows interquartile range with horizontal line in the box showing median (RA n = 17, OA n = 7) Kruskal- Wallis ANOVA was used followed by the Dunn’s multiple comparison test ^*^p = <0.05, ^**^p = <0.01, ^***^p = <0.001.

In serum, CCL7 was detected almost solely in RA, being present in the serum of only one OA (Fig. 3A). In the SF, CCL7 was present in the majority of RA but no OA patients. Further analysis showed significant differences between CCL7 levels in the RA and OA SF and OA serum (*p* *<* 0.01).

CCL14 was detected in the serum and SF of RA and OA at comparatively high levels (Fig. 3B). The results indicated no significant differences in CCL14 levels between RA and OA serum or SF.

As with CCL14, CCL16 was detected in both the serum and SF of RA and OA. However there was a significant difference (*p* < 0.001) between CCL16 levels in RA serum compared to RA SF and OA SF (Fig. 3C). There were no significant differences between RA serum and OA serum CCL16 levels observed.

CCL22 was detected in the serum and SF of RA and OA (Fig. 3D). The CCL22 level in the RA serum was shown to be significantly higher than in OA SF (*p* < 0.05).

### 3.4 Analysis of correlations in RA SF and serum

Spearman rank correlation analysis followed by the Holm-Bonferroni test on serum and SF showed a significant correlation between serum levels of CCL7 and CCL16 (R = 0.64, *p* = 0.016). A highly significant correlation between SF CCL7 levels and anti-citrullinated antibody (anti-CCP) levels was also observed (R = 0.93, *p* = 0.001). No significant correlations were observed between levels of CCCL7, CCL14, CCL16 or CCL22 and the clinical measures of disease activity, joint damage or function

## 4 Discussion

Inflammation under normal (non-RA) conditions is a protective mechanism in response to injury and/or tissue damage whereby leukocytes travel to the site of injury to remove infectious agents and facilitate tissue repair. The transendothelial migration of leukocytes is also a major feature in chronic inflammation in RA as it is the method by which leukocytes in the post capillary venules cross from the circulating blood to the site of ‘injury’. This migration across ECs and the basement membrane into the synovial tissue and SF is mediated via a range of processes, including the action of chemokines [5].

This study has compared the presence of 26 of the CC-family chemokines in RA synovial ECs. The detection of chemokines varied, the least abundant being CCL27 which was present in 8.3% of RA blood vessels and the most abundant being CCL19 which was present in 80%. Of the 26, 19 have not been previously observed in RA ECs (see Table 1, Table 2). However many of them, such as CCL12 [19] and CCL13 [20], [21] have been observed as being in the synovial tissue without analysing their exact localisation. From the 26 chemokines analysed in this study CCL4, CCL7, CCL8, CCL14, CCL16, CCL19 and CCL22 were present on ≥60% of vessels, of which CCL7, CCL14, CCL16 and CCL22 had not previously been identified as present in RA ECs.

Of the four most abundant novel EC chemokines found by this study, (CCL7, CCL14, CCL16 and CCL22), CCL14 showed the most significant increase in RA synovial ECs compared to non-RA. This is the first study to show CCL14 to be present in RA ECs and suggests that this chemokine may play a role in the recruitment of inflammatory cells into the RA synovium as it has been shown to chemoattract monocytes, eosinophils and T-cells [22].

CCL22 was also significantly increased in RA ECs compared to non-RA (*p* = 0.014). This relates to earlier work identifying CCL22 ‘scattered throughout’ RA synovium and CCR4, the CCL22 receptor, localised on ECs [23]. In the current study the increased presence of CCL22 in RA ECs indicates that it may have a role in the recruitment of inflammatory cells to the RA synovium such as monocytes and T lymphocytes.

Our study showed CCL7 to be highly expressed on RA ECs, however further examination showed no significant differences between RA and non-RA. This was also supported by work from a different group [20] who first identified CCL7 in RA tissue and found it to be ‘abundantly present’ in all arthritis and control groups tested. The presence of CCL7 on stromal cells resembling fibroblasts within the RA tissue is in agreement with Haringman et al. who showed marked expression by fibroblast-like synoviocytes (FLS) and macrophages [20].

While CCL16 has been previously identified in RA tissue [20], [24], [25] this is the first study to identify its presence in RA ECs. The results indicate that there are no significant differences between VWF+ ECs in RA and non-RA tissue and so it may be unlikely that CCL16 plays a dominant role in the elevated leukocyte trafficking into the synovium seen in RA.

The chemokines CCL4, CCL8 and CCL19 which have not been quantified by other studies but have been identified as present in RA ECs have also been further examined here. It was established that CCL19 was the most highly expressed at RA ECs, followed by CCL8 then CCL4. While CCL4 has been previously identified in RA ECs [26], [27] this study is the first to quantify its presentation in comparison to control non-RA tissue, where the control tissue is not from another arthritis type. The amount of CCL4 had previously been established as decreased in RA SF compared to OA SF [26]; the present study shows that CCL4 does not significantly differ in RA compared to non-RA ECs. While CCL4 acts as a chemoattractant for a variety of leukocytes including T-cells and B-cells [28] as well as monocytes and NK cells [29] the results of this study indicate that it may not be primarily important in stimulating the elevated leukocyte recruitment seen in RA.

CCL8 activates and chemoattracts a range of cells, including monocytes, T-cells, NK cells and fibroblast-like synoviocytes (FLS) [30], [31]. This study showed that the amount of CCL8 is significantly increased in RA ECs compared to non-RA ECs, which is supported by an earlier study where a significant increase of CCL8 in RA ECs was observed, but in comparison to OA ECs [32]. The presence of CCL8+ stromal cells which were fibroblast-like have also been observed in a previous report [20].

The presence of CCL19 in RA ECs agrees with earlier reports where CCL19 was established as present at RA ECs [33] and was shown to be expressed on both lymphatic and vascular ECs in RA [34]. The present study also suggested more intense CCL19 staining to be present in the more densely infiltrated lymphoid follicle infiltrates and ECs. This supports earlier work which found CCL19 to be expressed in RA tissue where germinal centres were present, and absent where only diffuse infiltrates were found [33]. Our results show that CCL19 was highly up-regulated on RA ECs compared to non-RA and supports the notion of it being an important chemokine in lymphocyte recruitment.

The presence of chemokines differentially expressed in synovial tissues raised the question of examining their presence in the SF and sera of RA and OA patients. It is accepted that certain chemokines are found in the serum and/or SF of arthritis sufferers and that the chemokine profiles in serum and/or SF differ with disease duration and between different arthritides. This may allow for the further identification of potential disease markers. For example, SF levels of cytokines in patients with early RA have been shown to have differing cytokine profiles at different disease stages [13], [14]. Chemokines found in the SF of RA patient such as CCL3 and CCL5 have been shown to be upregulated in RA compared to other arthropathies [35], [36].

The significant correlations between CCL7 levels in RA SF and anti-CCP antibodies in RA combined with the lack of CCL7 in OA SF suggests that CCL7 may be a novel marker for RA. The increase in SF CCL7 in RA may be due to CCL7 generation by the abundant macrophages of the RA joint [37], [38]. Furthermore, the presence of CCL7 in SF at significantly greater concentrations than in serum, indicates that the synovial tissue may be a primary source of CCL7.

We have shown that there are no significant differences in the CCL14 SF and serum levels in RA compared to OA. CCL14 has been shown to be expressed at high concentrations of 10 nM (86,730 pg/ml) in the plasma of healthy individuals which significantly increases up to 80nM (693,840 pg/ml) in patients with renal disease [39]. However, it is unusual for a chemokine to reach such levels, even in a disease state.

CCL16 was significantly higher in RA serum compared to matched SF, and to OA SF. This suggests that it may be generated remotely, mainly by other tissues or possibly other joints, rather than by the knee joints studied here. As with CCL14, CCL16 was shown to be present at relatively high concentrations. This is lower than chemokines such as CCL2, but higher than CCL5 in RA [38]. However, there were no significant CCL16 increases in RA serum compared to OA serum suggesting it may not be a potential marker in RA. Due to a lack of correlation between serum/SF levels and clinical variables it is unlikely to be of significance as a marker of disease activity or severity. Flytlie et al. [40] showed increased CCL22 levels in RA compared to OA and healthy plasma. The current study showed that CCL22 levels were significantly increased in RA serum compared to OA SF with no significant correlations with clinical variables evident.

Our data suggest that several chemokines are abundantly present at the ECs of RA synovium, rather than occurring individually. This suggests potential synergistic effects between the chemokines may promote disease. Furthermore, synergism has been observed in previous studies with a range of mechanisms having been put forward to account for this [41]. As it has been shown that CXCL13 and CCL21 have synergistic effects in lymphoid tissue production in RA synovitis [42] and more recently that CCL7 has been shown to synergise with CXCL8 in acute respiratory distress syndrome to promote neutrophil migration [43]. Thus it is possible that currently unexplored synergistic responses between CCL14, CCL19 and CCL22 for example and other chemokines are present in RA ECs.

## 5 Conclusions

The presence of 26 of the CC-chemokines in RA synovial ECs have been quantified and compared. The chemokines CCL7, CCL14, CCL16 and CCL22 were established as being present at RA synovial ECs for the first time. These early results show a significant increase of CCL8, CCL14, CCL19 and CCL22 in RA compared to non-RA synovium and following further validation may suggest that EC presentation of these chemokines could also play a role in the recruitment of inflammatory cells in RA. Further studies are needed to explore the functionality of a number of these EC chemokines and their potential roles in RA. The study also indicates that SF CCL7 may be a novel RA disease marker.

## Funding

The studentship which formed the basis of this PhD was contributed to by the Medical Research Council. Consumables costs for this work was supported by the Oswestry Rheumatology Association (Grant No. PG116, 2010)
