# Supplementary material for: An initial investigation into endothelial CC chemokine expression in the human rheumatoid synovium
Source: Cytokine. 2017 Sep;97:133–40. doi: 10.1016/j.cyto.2017.05.023 (PMC5516773; doi:10.1016/j.cyto.2017.05.023)
Supplement: Supplementary data 1 [file mmc1.docx]

**Supplementary data table 1 Showing solutions used for immunofluorescence** (continued on next 2 pages)

| **Chemokine under investigation** | **RA/non-RA Tissue** | **Blocking buffer** | ***Dilution**  **buffer** | **Primary antibodies and**  **concentrations used** | **Secondary antibodies and concentrations used** |
| --- | --- | --- | --- | --- | --- |
| **CCL1** | RA | A | A | Rabbit anti-human VWF, 3μg/ml  Mouse anti-human CCL1, 2.5μg/ml | Donkey anti-rabbit 594, 1μg/ml  Goat anti-mouse 488 IgM, 3.3μg/ml |
| **CCL2** | RA | C | C | Rabbit anti-human VWF, 3μg/ml  Mouse anti-human CCL2, 10μg/ml | Donkey anti-rabbit 488 IgG, 3.3μg/ml Goat anti-mouse 594 IgG_2b_, 6.6μg/ml |
| **CCL3** | RA | C | C | Rabbit anti-human VWF, 3μg/ml  Mouse anti-human CCL3, 10μg/ml | Goat anti-rabbit 488, 6.6μg/ml  Goat anti-mouse 594 IgG_1_, 3.3μg/ml |
| **CCL4** | RA/ NON-RA | D | B | Rabbit anti-human VWF, 3μg/ml  Goat anti-human CCL4 , 10μg/ml | Donkey anti-rabbit 488 IgG, 3.3μg/ml  Donkey anti-goat 594, 6.6μg/ml |
| **CCL5** | RA | D | B | Rabbit anti-human VWF, 3μg/ml  Goat anti-human CCL5 , 15μg/ml | Donkey anti-rabbit 488 IgG, 3.3μg/ml  Donkey anti-goat 594, 6.6μg/ml |
| **CCL7** | RA/ NON-RA | A | A | Mouse anti-human VWF, 4μg/ml  Rabbit anti-human CCL7, 2.5μg/ml | Donkey anti-mouse 594 IgG_1_, 3.3μg/ml  Donkey anti-rabbit 488 IgG, 3.3μg/ml |
|  | RA/NON-RA | A | A | Rabbit anti-human CCL7, 2.5μg/ml  Goat anti-human LYVE-1, 7.5μg/ml | Donkey anti-rabbit 488 IgG, 3.3μg/ml  Donkey anti-goat 594, 3.3μg/ml |
| **CCL8** | RA/NON-RA | A | A | Rabbit anti-human VWF, 3μg/ml  Goat anti-human CCL8, 2μg/ml | Donkey anti-goat 594, 6.6μg/ml  Donkey anti-rabbit 488 IgG, 3.3μg/ml |
| **CCL10** | RA | A | A | Mouse anti-human VWF, 4μg/ml  Rabbit anti-human CCL10, 4μg/ml | Goat anti-mouse 488, 3.3μg/ml  Donkey anti-rabbit 594, 6.6μg/ml |
| **CCL11** | RA | B | B | Rabbit anti-human VWF, 3μg/ml  Mouse anti-human CCL11, 15μg/ml | Donkey anti-rabbit 488 IgG, 3.3μg/ml  Donkey anti-mouse 594 IgG_1_, 6.6μg/ml |
| **CCL12** | RA | A | A | Mouse anti-human VWF, 4μg/ml  Rabbit anti-human CCL12, 2μg/ml | Goat anti-mouse 488, 3.3μg/ml  Donkey anti-rabbit 594, 6.6μg/ml |

**Supplementary data table 1 continued**

| **Chemokine under investigation** | **RA/NON-RA Tissue** | **Blocking buffer** | ***Dilution**  **buffer** | **Primary antibodies and**  **concentrations used** | **Secondary antibodies and concentrations used** |
| --- | --- | --- | --- | --- | --- |
| CCL13 | RA | A | A | Rabbit anti-human VWF, 3μg/ml  Goat anti-human CCL13, 4μg/ml | Donkey anti-goat 594, 6.6μg/ml  Donkey anti-rabbit 488 IgG, 3.3μg/ml |
| **CCL14** | RA/NON-RA | C | C | Rabbit anti-human VWF, 3μg/ml  Mouse anti-human CCL14, 2μg/ml | Donkey anti-rabbit 488 IgG, 3.3μg/ml  Goat anti-mouse 594, 2.5 μg/ml |
|  | RA/NON-RA | C | C | Mouse anti-human CCL14, 2μg/ml  Goat anti-human LYVE-1, 15μg/ml | Donkey anti-mouse 594, 6.6μg/ml  Donkey anti-rabbit 488 IgG, 3.3μg/ml |
| **CCL15** | RA | A | A | Mouse anti-human VWF, 4μg/ml  Rabbit anti-human CCL15, 4μg/ml | Goat anti-mouse 488, 3.3μg/ml  Donkey anti-rabbit 594, 6.6μg/ml |
| **CCL16** | RA/NON-RA | C | C | Rabbit anti-human VWF, 3μg/ml  Goat anti-human CCL16, 15μg/ml | Donkey anti-goat 488 , 3.3μg/ml  Donkey anti-rabbit 594, 6.6μg/ml |
|  | RA/NON-RA | C | C | Goat anti-human CCL16, 15μg/ml  Rabbit anti-human LYVE-1, 4μg/ml | Donkey anti-goat 488 , 3.3μg/ml  Donkey anti-rabbit 594, 3.3μg/ml |
| **CCL17** | RA | B | B | Rabbit anti-human VWF, 3μg/ml  Goat anti-human CCL17,10μg/ml | Donkey anti-rabbit 488 IgG, 3.3μg/ml  Donkey anti-goat 594, 6.6μg/ml |
| **CCL18** | RA | A | A | Rabbit anti-human VWF, 3μg/ml  Goat anti-human CCL18,4μg/ml | Donkey anti-rabbit 488 IgG, 3.3μg/ml  Donkey anti-goat 594, 6.6μg/ml |
| **CCL19** | RA/NON-RA | C | C | Rabbit anti-human VWF, 3μg/ml  Mouse anti-human CCL19, 20μg/ml | Donkey anti-rabbit 488 IgG, 3.3μg/ml  Goat anti-mouse 594, 6.6μg/ml |
| **CCL20** | RA | B | B | Rabbit anti-human VWF, 3μg/ml  Goat anti-human CCL20,100μg/ml | Donkey anti-rabbit 488 IgG, 3.3μg/ml  Donkey anti-goat 594, 6.6μg/ml |
| **CCL21** | RA | A | A | Rabbit anti-human VWF, 3μg/ml  Goat anti-human CCL21, 15μg/ml | Donkey anti-rabbit 488 IgG, 3.3μg/ml  Donkey anti-goat 594, 6.6μg/ml |

**Supplementary data table 1 continued**

| **Chemokine under investigation** | **RA/NON-RA Tissue** | **Blocking buffer** | ***Dilution**  **buffer** | **Primary antibodies and**  **concentrations used** | **Secondary antibodies and**  **concentrations used** |
| --- | --- | --- | --- | --- | --- |
| **CCL22** | RA/NON-RA | A | A | Rabbit anti-human VWF, 3μg/ml  Goat anti-human CCL22, 4μg/ml | Donkey anti-rabbit 488 IgG, 3.3μg/ml  Donkey anti-goat 594, 6.6μg/ml |
|  | RA/NON-RA | A | A | Goat anti-human CCL22, 4μg/ml  Rabbit anti-human LYVE-1, 4μg/ml | Donkey anti-goat 594 IgG, 3.3μg/ml  Donkey anti-rabbit 488, 6.6μg/ml |
| **CCL23** | RA | A | A | Rabbit anti-human VWF, 3μg/ml  Goat anti-human CCL23, 4μg/ml | Donkey anti-rabbit 488 IgG, 3.3μg/ml  Donkey anti-goat 594, 6.6μg/ml |
| **CCL24** | RA | B | B | Rabbit anti-human VWF, 3μg/ml  Goat anti-human CCL24,15μg/ml | Donkey anti-rabbit 488 IgG, 3.3μg/ml  Donkey anti-goat 594, 6.6μg/ml |
| **CCL25** | RA | A | A | Rabbit anti-human VWF, 3μg/ml  Goat anti-human CCL25, 2μg/ml | Donkey anti-rabbit 488 IgG, 3.3μg/ml  Donkey anti-goat 594, 6.6μg/ml |
| **CCL26** | RA | A | A | Rabbit anti-human VWF, 3μg/ml  Goat anti-human CCL26,15μg/ml | Donkey anti-rabbit 488 IgG, 3.3μg/ml  Donkey anti-goat 594, 6.6μg/ml |
| **CCL27** | RA | A | A | Rabbit anti-human VWF, 3μg/ml  Mouse anti-human CCL27, 20μg/ml | Donkey anti-rabbit 488 IgG, 2.8μg/ml  Goat anti-mouse 594, 5μg/ml |
| **CCL28** | RA | A | A | Rabbit anti-human VWF, 3μg/ml  Goat anti-human CCL28, 4μg/ml | Donkey anti-rabbit 488 IgG, 3.3μg/ml  Donkey anti-goat 594, 6.6μg/ml |

This table shows the primary and secondary antibody solutions used following the optimisation procedures.

*Dilution buffer - for secondary antibody solutions, 10% human serum was also included. All of the above experiments were carried out with isotype matched negative controls. The details of the buffers used were

Blocking buffer A – 10% donkey serum and 0.3% triton X in PBS.

Blocking buffer B – 2%BSA, 10% non-fat dry milk 0.3% triton X in PBS.

Blocking buffer C – PBS only.

Blocking buffer D –5% BSA, 10% non-fat dry milk 0.3% triton X in PBS

Dilution buffer A – 1% BSA, 1% donkey serum and 0.3% triton X in PBS

Dilution buffer B – 1%BSA, 1% non-fat dry milk 0.3% triton X in PBS

Dilution buffer C – PBS only

Dilution buffer D – 2% BSA, 10% non-fat dry milk 0.3% triton X in PBS
